# Microfluidic device facilitates in vitro modeling of human neonatal necrotizing enterocolitis–on-a-chip

**DOI:** 10.1172/jci.insight.146496

**Published:** 2023-04-24

**Authors:** Wyatt E. Lanik, Cliff J. Luke, Lila S. Nolan, Qingqing Gong, Lauren C. Frazer, Jamie M. Rimer, Sarah E. Gale, Raymond Luc, Shay S. Bidani, Carrie A. Sibbald, Angela N. Lewis, Belgacem Mihi, Pranjal Agrawal, Martin Goree, Marlie Maestas, Elise Hu, David G. Peters, Misty Good

**Affiliations:** 1 Washington University School of Medicine, St. Louis, Missouri, USA.; 2 University of North Carolina at Chapel Hill, Chapel Hill, North Carolina, USA.; 3 Emulate, Inc. Boston, Massachusetts, USA.; 4 Washington University in St. Louis, St. Louis, Missouri, USA.; 5 University of Pittsburgh and Magee-Womens Research Institute, Pittsburgh, Pennsylvania, USA.

**Keywords:** Cell Biology, Inflammation, Cytokines, Expression profiling, Tight junctions

## Abstract

Necrotizing enterocolitis (NEC) is a deadly gastrointestinal disease of premature infants that is associated with an exaggerated inflammatory response, dysbiosis of the gut microbiome, decreased epithelial cell proliferation, and gut barrier disruption. We describe an in vitro model of the human neonatal small intestinal epithelium (Neonatal-Intestine-on-a-Chip) that mimics key features of intestinal physiology. This model utilizes intestinal enteroids grown from surgically harvested intestinal tissue from premature infants and cocultured with human intestinal microvascular endothelial cells within a microfluidic device. We used our Neonatal-Intestine-on-a-Chip to recapitulate NEC pathophysiology by adding infant-derived microbiota. This model, named NEC-on-a-Chip, simulates the predominant features of NEC, including significant upregulation of proinflammatory cytokines, decreased intestinal epithelial cell markers, reduced epithelial proliferation, and disrupted epithelial barrier integrity. NEC-on-a-Chip provides an improved preclinical model of NEC that facilitates comprehensive analysis of the pathophysiology of NEC using precious clinical samples. This model is an advance toward a personalized medicine approach to test new therapeutics for this devastating disease.

## Graphical abstract

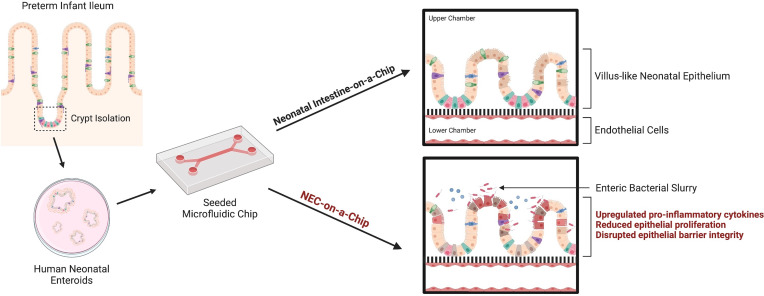

## Introduction

Necrotizing enterocolitis (NEC) is a severe, potentially fatal intestinal disease of prematurity that results in intestinal injury and necrosis (1). NEC is characterized by intestinal dysbiosis and an injurious inflammatory response (1–3). Central to the pathophysiology of NEC is intestinal epithelial cell damage that leads to a loss of intestinal barrier integrity in the setting of decreased epithelial cell proliferation and increased epithelial cell death (1–3). This compromised epithelial layer permits bacterial translocation across the gut barrier, which can result in sepsis. Despite intensive research with various models of the disease, the mechanisms underlying the pathophysiology NEC are incompletely understood, and thus, novel treatment approaches and new preventative strategies for NEC remain lacking. NEC research has thus far been hindered by the extremely limited nature of samples from preterm neonates due to the need for intestinal tissue to be harvested during a surgical procedure. In vitro modeling of NEC has the potential to accelerate this research; however, current in vitro NEC models do not accurately simulate the multiple cell types and complex intestinal dysbiosis that have been implicated in the disease. For example, rat intestinal epithelial (IEC-6) and human colon adenocarcinoma (Caco-2) cell lines are often used to study molecular pathways and responses to therapeutics in NEC (4–7). However, these monotypic epithelial cell lines do not fully recapitulate the premature intestinal cell architecture and complexity, restricting the physiologic relevance of these models (8).

Other in vitro models have used stem cells derived from human small intestinal crypts to produce enteroids (i.e., spheroids or organoids) (9–12). Intestinal organoid models can differentiate into every subtype of intestinal epithelial cell and have apical-basolateral polarity within a 3-dimensional (3D) spherical architecture (9, 13). However, due to their enclosed nature, the ability to study the effect of microbial interactions or therapeutics on the apical epithelial surface is limited. In contrast, a 2-dimensional (2D) enteroid monolayer system allows for direct exposure of the apical epithelium to bacteria and therapeutics (14). Despite this advantage, the bacteria in these static systems quickly overproliferate, causing exaggerated epithelial damage and rapid cell death, reducing the ability to model NEC effectively in vitro (15).

Recent advances in microfluidics technology have aided the development of in vitro modeling using organ-on-a-chip platforms (15). Intestine-on-a-chip models have been developed to closely simulate the microenvironment of human small intestine through comprehensive cellular differentiation, the formation of 3D villus-like axes, mucus production, continuous luminal flow, and the ability to mimic peristalsis by adding a stretch component (16, 17).

In this study, we describe the development of an in vitro model of a premature infant–derived Neonatal-Intestine-on-a-Chip microphysiological platform to model the interactions between the intestinal epithelium, endothelium, and microbiome. Furthermore, we adapted this Neonatal-Intestine-on-a-Chip model to simulate the pathophysiology of the human disease NEC. This model, named NEC-on-a-Chip, utilizes a combination of premature infant small intestinal enteroids cocultured with human intestinal microvascular endothelial cells and patient-derived microbiota to recreate critical features of premature gut pathophysiology, including microbial dysbiosis. This improved in vitro model system could accelerate the advancement of potential therapeutics for NEC and serve as a tool in precision medicine by the generation of patient-specific treatments.

## Results

### Neonatal-Intestine-on-a-Chip models human small intestinal architecture.

**Figure 1:**
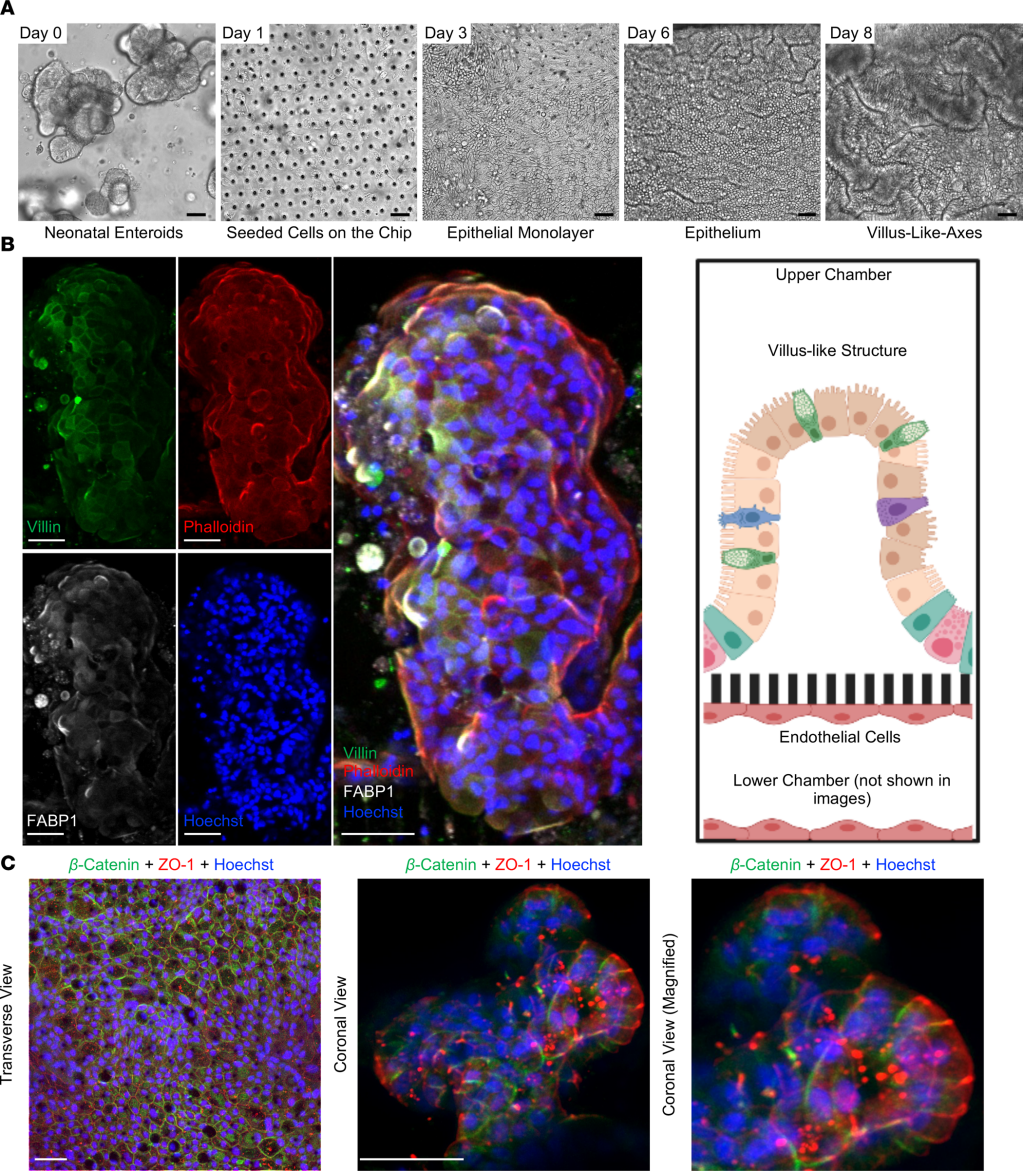
Development of the Neonatal-Intestine-on-a-Chip microfluidic model. (**A**) Growth progression of Neonatal-Intestine-on-a-Chip is shown by brightfield microscopy, beginning with neonatal enteroids on day 0, seeding of stem cells within a microfluidic device on day 1, development of a confluent monolayer by day 3, invaginations of epithelium through day 6, and advancement to villus-like-axes on day 8. Scale bars: 50 μm. Images representative of more than 20 independent experiments. (**B**) Left: Representative immunofluorescence images of a deconvoluted coronal cross section of a villus-like formation within the Neonatal-Intestine-on-a-Chip is shown separated and merged (merged) with villin (green, microvilli brush border), phalloidin (red, actin), FABP1 (white, enterocytes), and Hoechst (blue, nuclei). Scale bars: 50 μm. Right: Illustration demonstrating coronal view seen in immunofluorescence images (created with BioRender.com). (**C**) Representative immunofluorescence images of Neonatal-Intestine-on-a-Chip epithelium stained for β-catenin (green, basolateral component of adherens junctions), ZO-1 (red, apical tight junctions), and with Hoechst (blue). Scale bars: 50 μm.

We developed a Neonatal-Intestine-on-a-Chip model to recreate the microenvironment of the newborn small intestine in vitro. For this model, enteroids were differentiated from a surgically harvested piece of small intestine that was resected from a neonate with a noninflammatory intestinal condition. To establish a baseline for cellular growth, we monitored developmental progression of intestinal stem cells as enteroids (day 0) to physiologic neonatal intestinal epithelium (day 8) by brightfield microscopy (Figure 1A). On day 0, neonatal enteroids appear as discrete organoids. These enteroids were then dissociated and seeded into the Matrigel-coated microfluidic device (day 1). A confluent monolayer was established by day 3, followed by the appearance of furrows within the epithelium (day 6), and finally advancement to 3D architecture (day 8) with villus-like structures reminiscent of those found in vivo (Figure 1A).

To determine whether these villus-like structures developed architecture similar to the human small intestine, the cells within the microfluidic device were immunostained for 2 molecular markers of intestinal villi: villin, an intestinal brush border marker (green) and fatty acid binding protein 1 (FABP1), an enterocyte marker (white; Figure 1B). The villi were counterstained with phalloidin (actin, red) and Hoechst 33342 dye (nuclei, blue; Figure 1B). The architecture of these structures was remarkably similar to a human intestine, with development of a 3D villus-like structure lined by enterocytes with microvilli and an underlying actin support structure.

To determine the apical-basolateral polarity within the Neonatal-Intestine-on-a-Chip, cells were immunostained with primary antibodies against the apical tight junction marker, zonula occludens 1 (ZO-1, red), and a basolateral protein that is an important component of adherens junctions, β-catenin (green; Figure 1C). Confocal microscopy demonstrated that the ZO-1 marker was concentrated toward the lumen of the Neonatal-Intestine-on-a-Chip, whereas β-catenin was localized toward the semipermeable membrane (Figure 1C). These data suggest that our Neonatal-Intestine-on-a-Chip exhibits many of the central characteristics of the neonatal intestine, including 3D cellular architecture with villus-like formations, appropriate cell-cell adhesions, and epithelial cell polarity.

### Gene expression characterization of NEC-on-a-Chip.

**Figure 2:**
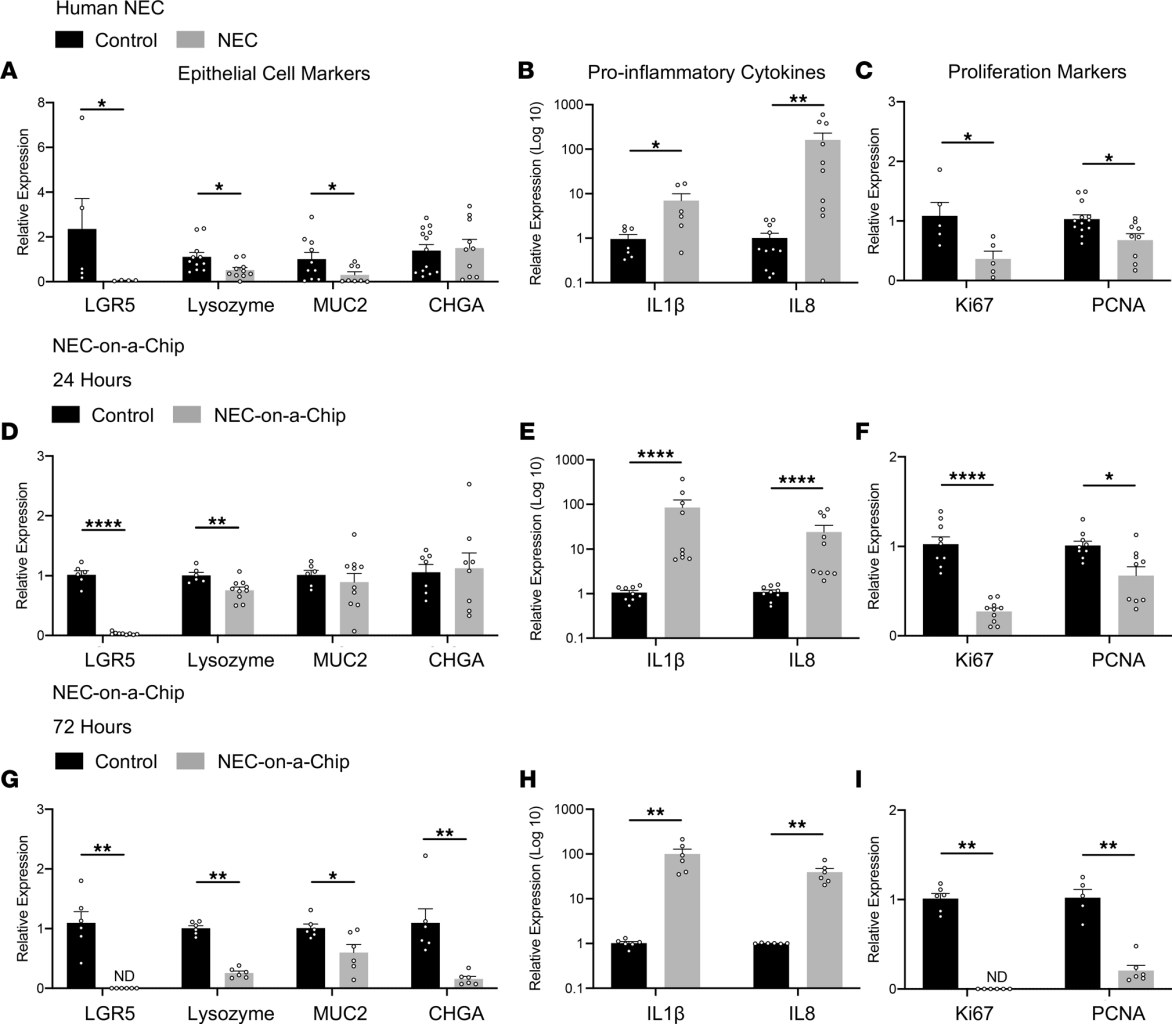
Epithelial cell–associated gene expression is reduced and inflammatory gene expression is increased in human NEC and NEC-on-a-Chip. (**A**–**C**) Human NEC. Relative gene expression in neonatal terminal ileal samples from infants with and without NEC were analyzed by qRT-PCR. (**A**) Specialized epithelial cell markers *LGR5* (Control *n* = 5; NEC *n* = 4), *LYZ* (Control *n* = 11; NEC *n* = 10), *MUC2* (Control *n* = 10; NEC *n* = 8), and *CHGA* (Control *n* = 13; NEC *n* = 10). (**B**) Proinflammatory cytokines *IL1B* (Control *n* = 7; NEC *n* = 6) and *IL8* (Control n=11; NEC *n* = 10). (**C**) Proliferation markers *Ki67* (Control *n* = 4; NEC *n* = 5) and *PCNA* (Control *n* = 13; NEC *n* = 9). Dots represent individual patient samples. (**D**–**F**) Relative gene expression for control chips versus NEC-on-a-Chip at 24 hours determined via qRT-PCR. (**D**) Specialized epithelial cell markers *LGR5* (Control *n* = 6; NEC-on-a-Chip *n* = 9), *LYZ* (Control *n* = 6; NEC-on-a-Chip *n* = 10), *MUC2* (Control n=6; NEC-on-a-Chip *n* = 10), and *CHGA* (Control *n* = 7; NEC-on-a-Chip *n* = 8). (**E**) Proinflammatory cytokines *IL1B* (Control *n* = 9; NEC-on-a-Chip *n* = 9) and *IL8* (Control *n* = 9; NEC-on-a-Chip *n* = 10). (**F**) Proliferation markers *Ki67* (Control *n* = 9; NEC-on-a-Chip *n* = 10) and *PCNA* (Control *n* = 9; NEC-on-a-Chip *n* = 9). Dots represent individual chips. (**G**–**I**) Relative gene expression for control chips versus NEC-on-a-Chip at 72 hours determined via qRT-PCR. (**G**) *LGR5* (Control *n* = 6; NEC-on-a-Chip *n* = 6), *LYZ* (Control *n* = 6; NEC-on-a-Chip *n* = 6), *MUC2* (Control *n* = 6; NEC-on-a-Chip *n* = 6), and *CHGA* (Control *n* = 6; NEC-on-a-Chip *n* = 6). (**H**) Proinflammatory cytokines *IL1B* (Control *n* = 6; NEC-on-a-Chip *n* = 6) and *IL8* (Control *n* = 6; NEC-on-a-Chip *n* = 6). (**I**) Proliferation markers *Ki67* (Control *n* = 6; NEC-on-a-Chip *n* = 6) and *PCNA* (Control *n* = 5; NEC-on-a-Chip *n* = 6). *n* = number of chips. Dots represent individual chips. Bars represent mean ± SEM. *P < 0.05, **P < 0.01, ****P < 0.0001 by Mann-Whitney *U* test. ND, not detected.

The goal of this system is to model the in vivo intestinal environment as closely as possible. Thus, we first compared gene expression in intestinal samples surgically isolated from neonates with NEC or from those undergoing bowel resection for noninflammatory conditions (control). We examined epithelial subtype markers and found that small intestinal tissue from neonates with NEC demonstrated decreased expression of stem cell (leucine-rich repeat–containing G protein–coupled receptor 5, *LGR5*), Paneth cell (lysozyme, *LYZ*), and goblet cell markers (*MUC2*) compared with control tissue (Figure 2A). There was no significant alteration in the expression of the enteroendocrine cell marker, chromogranin A (*CHGA*), in our cohort of patients (Figure 2A). Transcriptional analysis of proinflammatory cytokines demonstrated that both interleukin 1β (*IL1B*) and *IL8* were upregulated in intestinal tissue from neonates with NEC compared with controls (Figure 2B). Furthermore, we observed a significant reduction in the expression of proliferation markers *Ki67* and proliferating cell nuclear antigen (*PCNA*) in NEC tissues when compared with control samples (Figure 2C). These expression patterns are congruent with previous reports in the literature describing reduced numbers of specialized epithelial cells and impaired epithelial cell proliferation during NEC (4, 5, 18–21).

The pathophysiology of NEC is multifactorial and incompletely defined. Intestinal bacteria and microbial dysbiosis have been repeatedly associated with disease (22, 23). In addition, intestinal epithelial cell damage and an unregulated inflammatory cycle result in impaired epithelial barrier integrity in the setting of epithelial cell death and inadequate epithelial cell proliferation (4). To model these processes in vitro*,* we added a layer of complexity to our Neonatal-Intestine-on-a-Chip model by adding the intestinal microbiome isolated from the intestine of a premature infant that died from severe NEC (24). This model, referred to as NEC-on-a-Chip, consists of the microfluidic device seeded with human neonatal enteroids from a neonate with a noninflammatory intestinal condition (as described above), as well as endothelial cells and intestinal bacteria. Comparison of gene expression patterns between chips with added intestinal bacteria (NEC-on-a-Chip) and those without bacteria (control) revealed significantly downregulated mRNA expression of crypt-associated epithelial cell markers *LGR5* and *LYZ* at 24 and 72 hours following introduction of the intestinal bacteria (Figure 2, D and G). At 24 hours, there was no significant difference in the gene expression of intestinal epithelial cell markers *MUC2* and *CHGA* (Figure 2D); however, these markers were significantly downregulated at 72 hours (Figure 2G). The bacteria–epithelial cell coculture in our NEC-on-a-Chip model resulted in increased expression of proinflammatory cytokines *IL1B* and *IL8* (Figure 2, E and H), as well as decreased expression of proliferation markers *Ki67* and *PCNA* (Figure 2, F and I) at both 24 and 72 hours after inoculation compared with control chips. Taken together, these data demonstrate the similarities between epithelial cell and inflammatory gene expression in NEC-on-a-Chip and human NEC.

**Figure 3:**
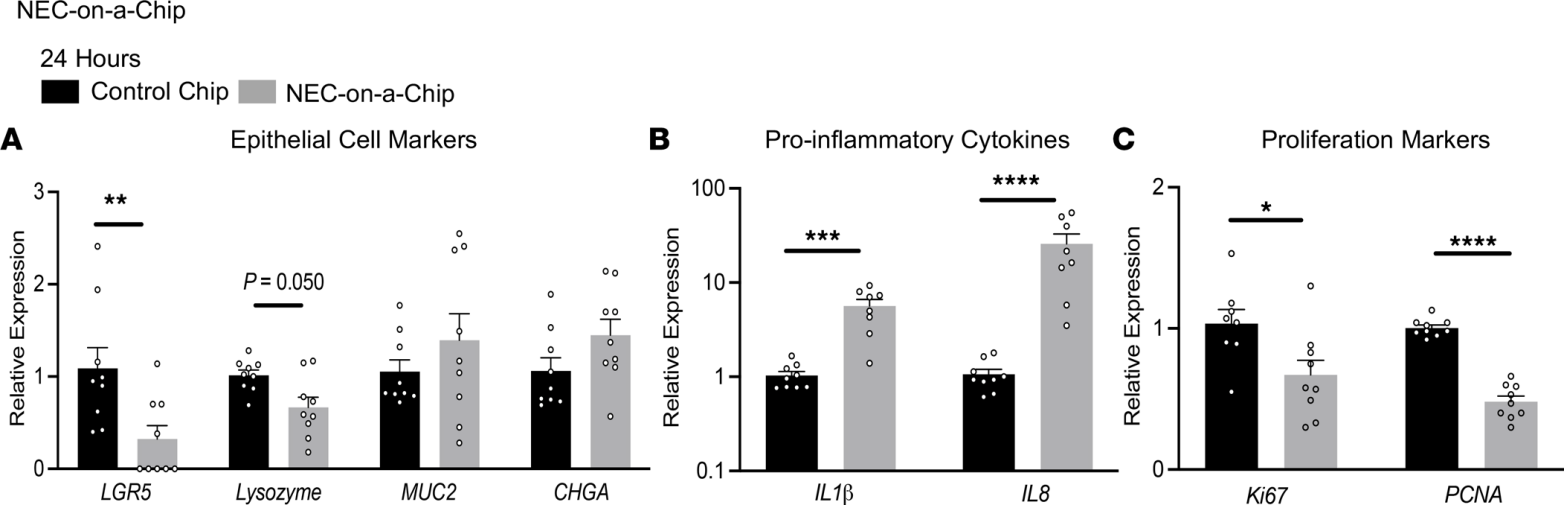
Epithelial cell–associated gene expression is reduced and inflammatory gene expression is increased in NEC-on-a-Chip using intestinal samples from neonates with NEC. Relative gene expression for control chips versus NEC-on-a-Chip at 24 hours determined via qRT-PCR. (**A**) Specialized epithelial cell markers *LGR5* (Control *n* = 9; NEC-on-a-Chip *n* = 9), *LYZ* (Control *n* = 9; NEC-on-a-Chip *n* = 9), *MUC2* (Control *n* = 9; NEC-on-a-Chip *n* = 9), and *CHGA* (Control *n* = 9; NEC-on-a-Chip *n* = 9), (**B**) Proinflammatory cytokines *IL1B* (Control *n* = 9; NEC-on-a-Chip *n* = 8) and *IL8* (Control *n* = 9; NEC-on-a-Chip *n* = 8). (**C**) Proliferation markers *Ki67* (Control *n* = 8; NEC-on-a-Chip *n* = 9) and *PCNA* (Control *n* = 9; NEC-on-a-Chip *n* = 9). *n* = number of chips. Samples are from 3 patients with NEC with data from 2–3 chips per patient. Dots represent individual chips. Bars represent mean ± SEM. *P < 0.05, **P < 0.01, ***P < 0.001, ****P < 0.0001 by Mann-Whitney *U* test.

One of the benefits of the NEC-on-a-Chip model is that fresh intestinal tissue from neonates with NEC is not required as a starting material for each model. Tissue from neonates with NEC is very limited in availability, and often, by the time of intestinal resection, the tissue available for research studies has variable viability. We are fortunate to have access to samples surgically resected from neonates with NEC, and we repeated the above gene expression experiments using enteroids generated from the intestinal tissue of 3 of these neonates (Figure 3). Changes in gene expression patterns in the presence (NEC-on-a-Chip) and absence of intestinal bacteria (control) were nearly identical to those observed at 24 hours for chips seeded with enteroids derived from an infant without NEC (Figure 2, D–F). In the NEC-on-a-Chip model, expression of the crypt-associated epithelial cell marker *LGR5* was significantly downregulated at 24 hours, and there was a trend toward significance for *LYZ* (Figure 3A). There were no significant differences in *MUC2* and *CHGA* expression between NEC-on-a-Chip and control chips at this time point (Figure 3A). We again demonstrated that the bacteria–epithelial cell coculture in our NEC-on-a-Chip model resulted in increased expression of proinflammatory cytokines *IL1B* and *IL8* (Figure 3B), as well as decreased expression of proliferation markers *Ki67* and *PCNA* (Figure 3C) at 24 hours after inoculation compared with control chips.

### NEC-on-a-Chip recapitulates several epithelial features seen in human NEC.

**Figure 4:**
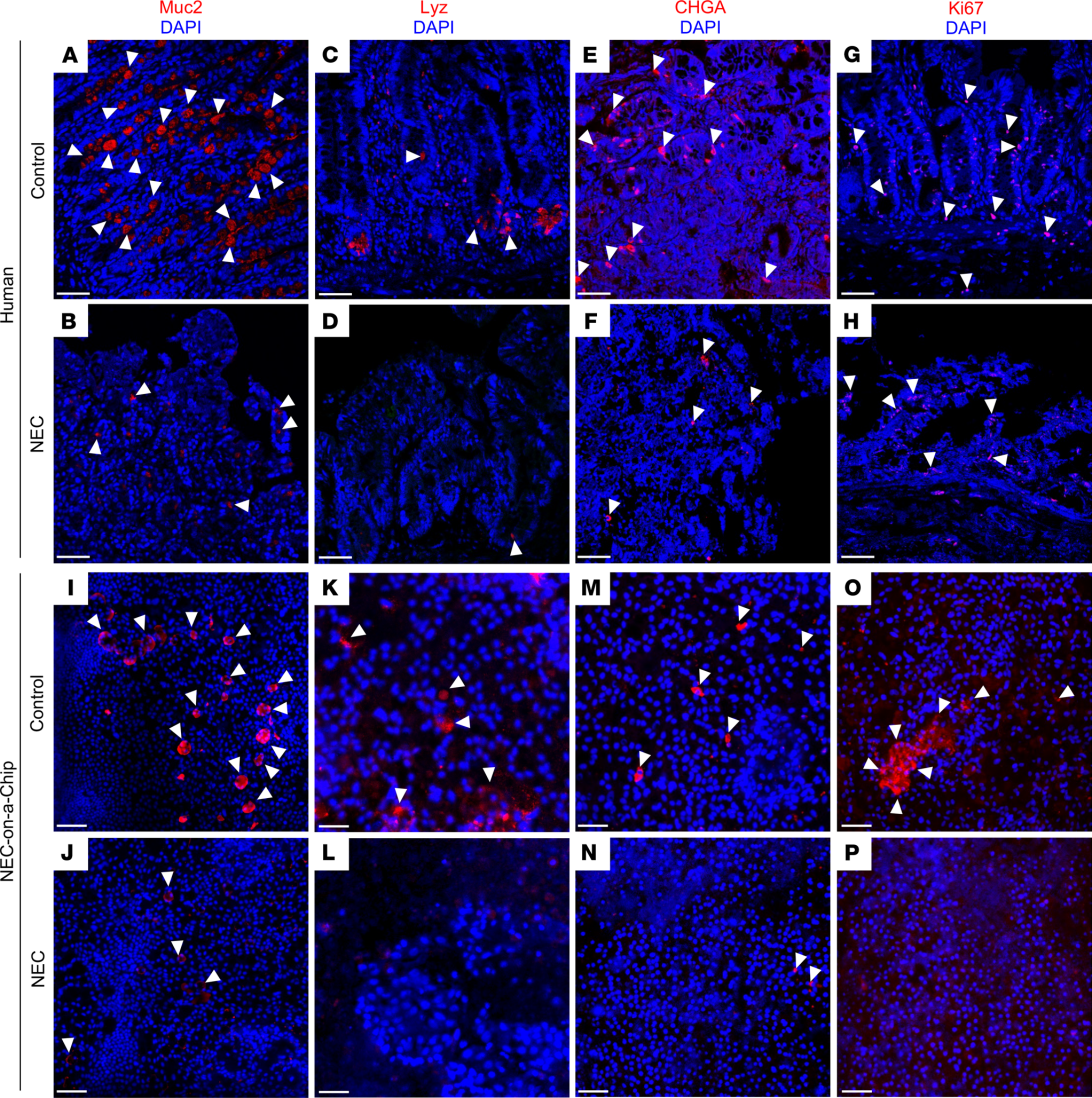
NEC-on-a-Chip recapitulates features of human NEC. Immunostaining of human control versus NEC ileum (first 2 rows) and Neonatal-Intestine-on-a-Chip (control) versus NEC-on-a-Chip (last 2 rows) for the goblet cell marker, Muc2 (red; **A**, **B**, **I**, and **J**), Paneth cell marker lysozyme (Lyz, red; **C**, **D**, **K**, and **L**), chromogranin A (CHGA, red; **E**, **F**, **M**, and **N**), and the proliferation marker Ki67 (red; **G**, **H**, **O**, and **P**). White arrows denote representative areas of positive staining. Scale bars: 50 μm.

The remainder of the experiments described in this study utilized enteroids derived from a patient with a noninflammatory intestinal condition, as described in Figures 1 and 2. Our next step was to utilize confocal microscopy to compare the changes in immunofluorescent staining of epithelial cell populations seen in human NEC with the NEC-on-a-Chip model 24 hours after introduction of intestinal bacteria. In both the human NEC and NEC-on-a-Chip models, there was a decrease in the number of goblet (MUC2; Figure 4, B and J), Paneth (lysozyme; Figure 4, D and L), and enteroendocrine cells (CHGA; Figure 4, F and N) when compared with their respective controls of healthy neonatal intestinal tissue (Figure 4, A, C, E, and G) and the Neonatal-Intestine-on-a-Chip (Figure 4, I, K, M, and O), respectively. Confocal microscopy also demonstrated reduction of the proliferation marker Ki67 in both human NEC tissue samples and NEC-on-a-Chip (Figure 4, H and P) compared with their respective controls (Figure 4, G and O). We have also previously described these patterns of specialized epithelial cell loss and reduced epithelial cell proliferation in the intestine of humans and mice with NEC (4, 11, 18). These findings demonstrate that the epithelial changes we and others have repeatedly observed during NEC can be recapitulated by our NEC-on-a-Chip model.

**Figure 5:**
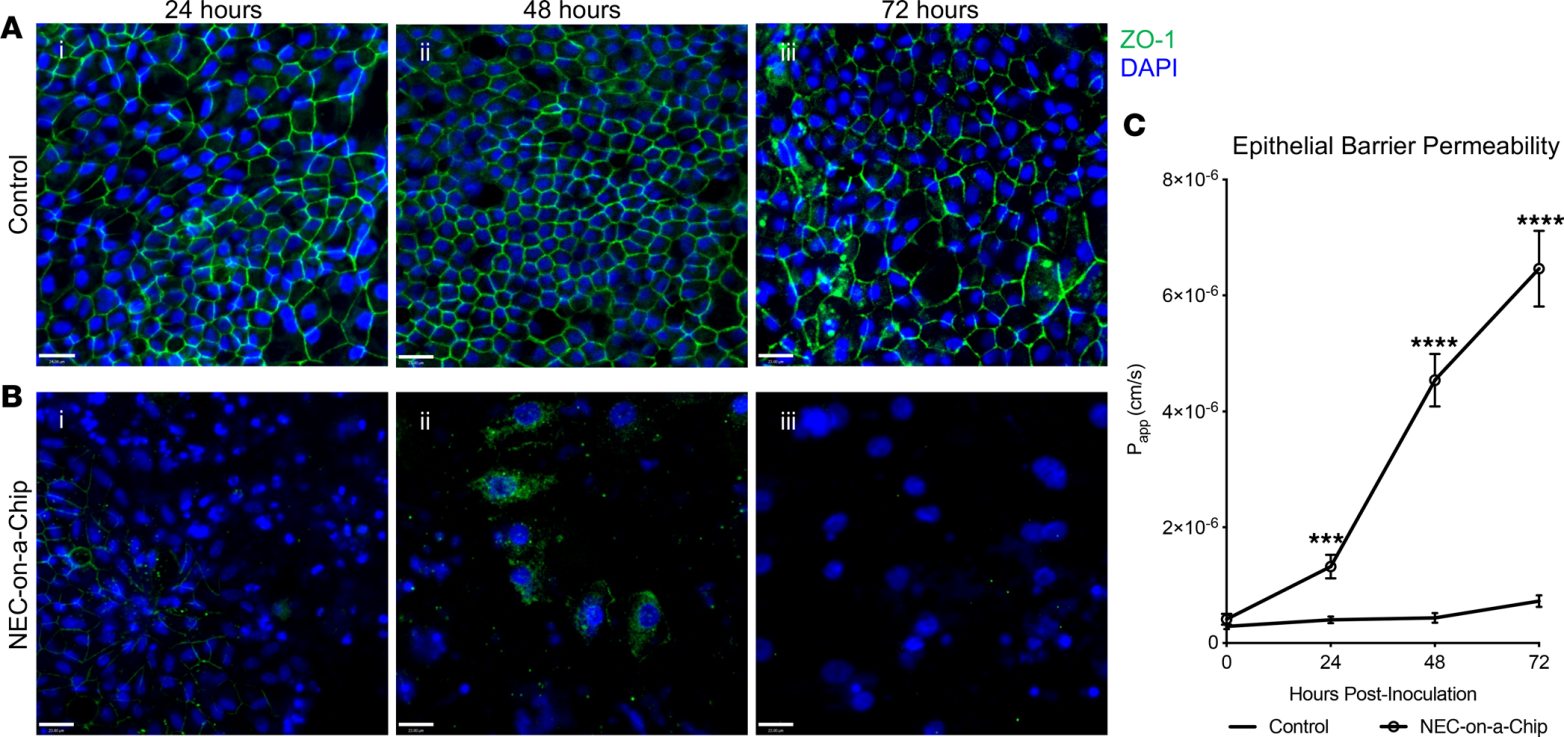
NEC-on-a-Chip has an impaired epithelial barrier. Immunofluorescent staining of tight junction marker ZO-1 (green) in (**A**) control chips and (**B**) NEC-on-a-Chip. Nuclei are stained with DAPI (blue). (**C**) Apparent paracellular permeability (P_app_) was assessed at 0 (*n* = 23), 24 hours (Control *n* = 12; NEC-on-a-Chip *n* = 12), 48 hours (Control *n* = 6; NEC-on-a-Chip *n* = 6), and 72 hours (Control *n* = 6; NEC-on-a-Chip *n* = 6). *n* = number of chips. Data represent mean ± SEM. ***P < 0.001, ****P < 0.0001 by 2-tailed, unpaired *t* test. Scale bars: 50 μm.

We next investigated the effects of microbial dysbiosis on the maintenance of epithelial tight junctions in our NEC-on-a-Chip model. Immunofluorescent staining for the epithelial tight junction marker ZO-1 showed decreased expression at 24, 48, and 72 hours when compared with controls (Figure 5, A and B). To characterize the degree of epithelial barrier dysfunction in the NEC-on-a-Chip model, we calculated the apparent paracellular permeability (P_app_), a measure of in vitro epithelial barrier permeability. The NEC-on-a-Chip model demonstrated increasing permeability over 72 hours following intestinal bacteria exposure, while the control chips showed no change (Figure 5C). These data suggest that we can model the progressive gut barrier dysfunction and failure seen in human NEC in our NEC-on-a-Chip model.

### Gene expression profiling in NEC-on-a-Chip mimics key pathways induced in human NEC.

**Figure 6:**
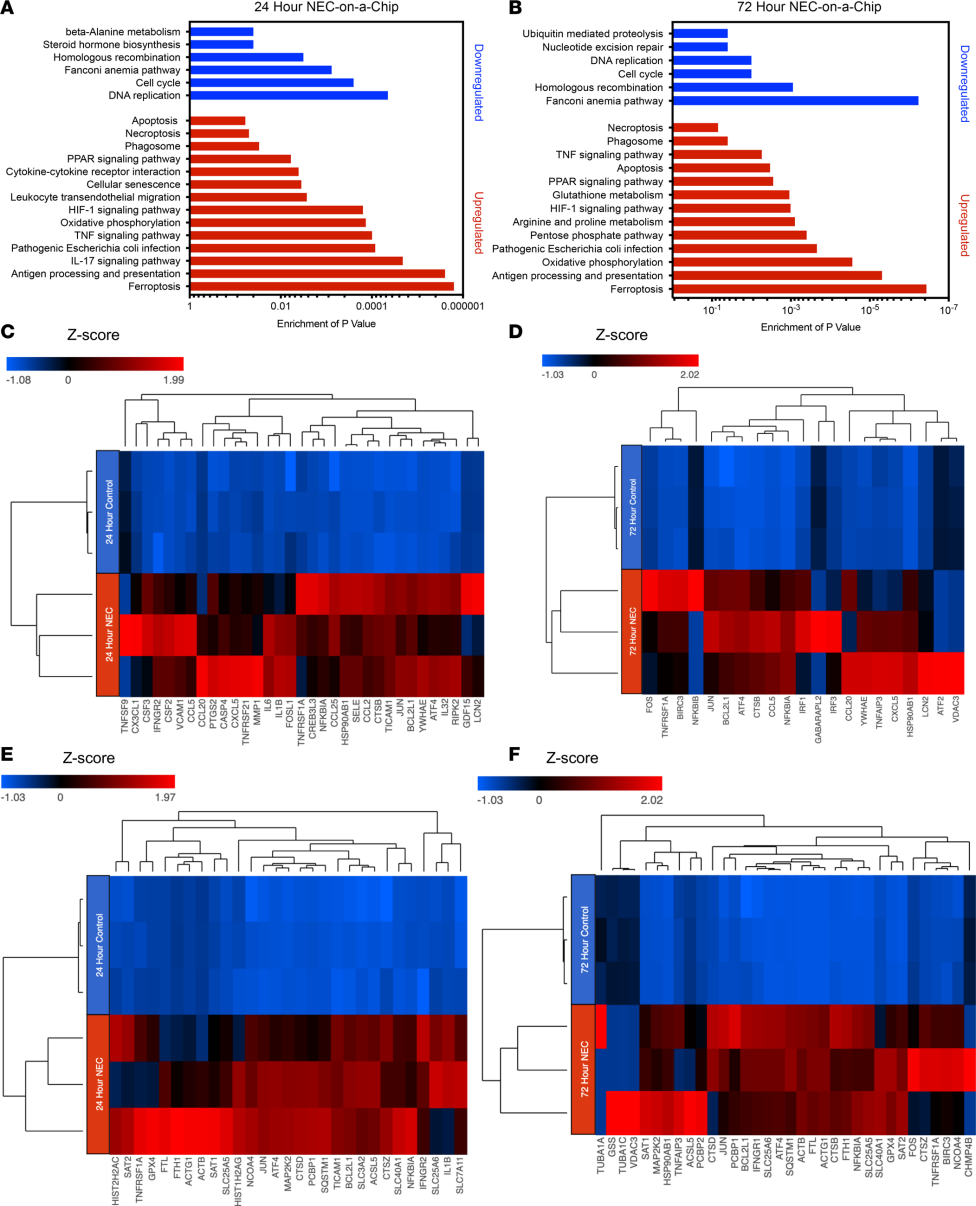
NEC-on-a-Chip contains a distinct profile of gene expression. Functional enriched pathways provide a comparative analysis of NEC-on-a-Chip (*n* = 3 chips) compared with controls (*n* = 3 chips) at (**A**) 24 and (**B**) 72 hours. Heatmap representation of targeted genes in NEC-on-a-Chip reveals the most significantly upregulated genes in proinflammatory pathways at (**C**) 24 and (**D**) 72 hours and in pathways related to cellular death at (**E**) 24 and (**F**) 72 hours.

To investigate the gene expression profile for NEC-on-a-Chip, we performed RNA-sequencing (RNA-seq) analysis on the small intestine epithelial cells in the NEC-on-a-Chip compared with control chips. A comparison of NEC-on-a-Chip with control chips at 24 hours identified a total of 3354 significantly upregulated and downregulated protein-coding genes. An additional comparison at 72 hours revealed a total of 9515 significantly expressed protein-coding genes. Figure 6, A and B identifies the most significant pathways modulated in the NEC-on-a-Chip after 24 and 72 hours, respectively. In correlation with the observed increased proinflammatory cytokine mRNA expression shown in Figure 2, D and G, the RNA-seq data demonstrated upregulated pathways related to inflammation, including the IL-17 signaling pathway, TNF signaling pathway, and cytokine–cytokine receptor interactions at 24 and 72 hours (Figure 6, A and B). The heatmap in Figure 6C displays the 33 most significantly upregulated genes related to proinflammatory pathways in the 24-hour NEC-on-a-Chip compared with the control chips, which includes chemokines, as well as the cytokines IL-1β and IL-6. Figure 6D reveals the 34 most significantly upregulated genes related to proinflammatory pathways in the 72-hour NEC-on-a-Chip when compared with controls.

Importantly, the RNA-seq analysis also revealed significant upregulation of genes involved in cellular death pathways (Figure 6, E and F). At 24 hours, we observed a significant upregulation of genes found in the ferroptosis pathway (*P* < 0.0001) in the NEC-on-a-Chip relative to control chips (Figure 6E), whereas at 72 hours, significant upregulation of the genes involved in the ferroptosis (*P* < 0.0001) and apoptosis (*P* < 0.005) cell death pathways were observed in the NEC-on-a-Chip when compared with controls (Figure 6F). The heatmap in Figure 6E displays the 29 most significant genes in the apoptosis, ferroptosis, and necroptosis pathways in the 24-hour comparison, and the heatmap in Figure 6F reveals the 34 most significant genes in these cell death pathways in the 72-hour comparison.

## Discussion

In this study, we describe an in vitro model of the neonatal intestinal epithelium that we have termed Neonatal-Intestine-on-a-Chip. Similar to previous adult and pediatric Intestine-on-a-Chip models (16, 17), the Neonatal-Intestine-on-a-Chip overcomes many limitations of traditional 2D and 3D intestinal cell culture techniques. Innovative in vitro models are needed to improve our understanding of human neonatal pathophysiology given the extremely limited availability of clinical samples and the difficulties associated with performing clinical trials in this fragile population.

The Neonatal-Intestine-on-a-Chip produces villus-like 3D architecture with robust epithelial cell differentiation into goblet, Paneth, and enteroendocrine cells in similar frequencies to those observed in the intestine of a premature infant. Epithelial polarity is preserved as evidenced through staining of the apical tight junctions for ZO-1 and basolateral proteins for β-catenin. The apical epithelial surface is easily accessible for exposure to experimental stimuli, such as bacteria or treatment with therapeutics. Additionally, the dynamic luminal flow with simulated peristaltic-like movements mimics intestinal peristalsis seen in humans. These features make the Neonatal-Intestine-on-a-Chip model an improved in vitro model of the premature intestinal microenvironment.

The Neonatal-Intestine-on-a-Chip was inoculated with enteric bacteria from a neonate with severe NEC to mirror the intestinal dysbiosis seen in NEC. This NEC-on-a-Chip model system accurately simulates several key features of the neonatal disease, including exaggerated inflammatory cytokine production, decreased specialized epithelial cell populations, reduced epithelial cell proliferation, and increased gut barrier permeability. Similar to the proinflammatory signature of human NEC, the NEC-on-a-Chip model demonstrated increased expression of *IL1B* and *IL8* at both 24 and 72 hours following inoculation with the intestinal bacteria. Additionally, RNA-seq analysis of the NEC-on-a-Chip epithelium found increased expression of several cytokines, chemokines, and inflammatory markers, including *IL1B*, *IL6*, *CXCL5,* and lipocalin 2 (*LCN2*). These findings suggest a proinflammatory microenvironment in NEC-on-a-Chip that is comparable to human NEC.

The proinflammatory environment seen in human NEC is associated with significant epithelial cell death and decreased epithelial cell populations. RNA-seq analysis showed upregulation of genes involved in the apoptosis, ferroptosis, and necroptosis cell death pathways in the NEC-on-a-Chip compared with controls. The NEC-on-a-Chip model also demonstrated decreased mRNA expression of the stem cell marker *LGR5* by 24 hours and complete loss of expression by 72 hours, reflecting the trends seen in human NEC tissue from both this and previously reported studies (18, 19). Paneth cell (lysozyme), goblet cell (MUC2), and enteroendocrine cell (CHGA) populations were decreased in both human NEC tissue and NEC-on-a-Chip. This is consistent with previous NEC literature demonstrating a decrease in these cell populations during the disease (25–27).

The role of enteroendocrine cells in NEC pathogenesis is not well established. Our evaluation of human tissue samples showed no difference in mRNA expression; however, there was an apparent decrease in the CHGA immunofluorescent staining between NEC and control samples. Immunofluorescent staining for CHGA in NEC-on-a-Chip at 24 hours revealed an observable difference between NEC-on-a-Chip and controls. Furthermore, the NEC-on-a-Chip model did show a decrease in *CHGA* mRNA expression at the 72-hour time point. Additional studies will be needed to look for the presence of late enteroendocrine cell injury in NEC and elucidate the physiologic significance of this phenomenon.

Our NEC-on-a-Chip model also accurately reflected the decreased cell proliferation seen in the neonatal disease (4, 5, 18–20). The mRNA expression of both *Ki67* and *PCNA* proliferation markers were decreased in human NEC tissue and NEC-on-a-Chip compared with controls. Immunofluorescent staining confirmed decreased Ki67 in both human NEC tissue and NEC-on-a-Chip compared with controls, indicating a decreased capacity for epithelial proliferation.

The loss of tight junctions in NEC contributes to a leaky gut barrier (21, 28), allowing bacterial translocation and increasing the risk of sepsis and death (18, 29, 30). Previous studies have found that *ZO1* mRNA expression is decreased in NEC tissue (21, 28, 31). We also demonstrated a decrease in the expression of the tight junction ZO-1 as well as its internalization by confocal microscopy and further demonstrated that the apparent paracellular permeability of the NEC-on-a-Chip model system increases with time.

There are inherent limitations in using in vitro models to study a complex in vivo process such as NEC. A visible 3D villus-like structure was formed on this microfluidic device, but these structures lack elements of complexity associated with intestinal villi in vivo such as immune cells, lymphatics, and systemic circulation. This model was also performed under aerobic conditions and thus lacks the influence of anaerobic conditions that are present in the human intestine. It is possible that anaerobic microenvironments form within the crypt/villus-like structure that forms on these chips. Lastly, the epithelium that grows in this monolayer inevitably changes in gene and protein expression signatures in the setting of in vitro culture. Unfortunately, surgical resections are not available in adequate abundance to perform mechanistic studies on directly isolated epithelial cells, and epithelial cells must be expanded in culture for further use. Therefore, recapitulating the in vivo intestinal environment as closely as possible via in vitro models is crucial to advancing this field of research.

The NEC-on-a-Chip model parallels many key in vivo features of NEC. It is a practical in vitro platform for studying NEC pathophysiology and testing candidate therapeutics. Since this model can be produced with patient-specific epithelium, it could be the first step toward developing a personalized medicine approach to study and treat NEC.

## Methods

### In vitro culture of human neonatal enteroids.

De-identified human ileal tissue was collected from neonates undergoing required resection in accordance with Washington University School of Medicine in St. Louis approved guidelines and regulations. Samples were obtained from infants undergoing surgical resection for NEC or for noninflammatory conditions as indicated. Tissue was processed as previously described in VanDussen et al. (32). In brief, neonatal small intestinal tissue was washed with Dulbecco’s Modified Eagle’s Medium/F12 (DMEM/F12, Invitrogen) with 4-(2-hydroxyethyl)-1-piperazineethanesulfonic acid (HEPES, Corning) supplemented with 10% heat-inactivated fetal bovine serum (Sigma-Aldrich), 2 mM L-glutamine (Gibco), 100 U/mL penicillin, and 0.1 mg/mL streptomycin (Sigma-Aldrich) to inactivate endogenous proteases. The tissue was digested with collagenase I, mechanically dissociated to release crypts, filtered, washed, and centrifuged for crypt isolation. Crypts were suspended in Matrigel growth factor–reduced matrix (Corning), which was polymerized at 37°C for 20 minutes. Enteroids were cultured in enteroid growth media consisting of a 50% vol/vol mixture of Advanced DMEM/F12 (Invitrogen) and conditioned media from an L cell line producing Wnt, R-spondin, and Noggin ligands (L-WRN; American Type Culture Collection, CRL-3276) (33) supplemented with 10 μM Y-27632 (R&D Systems), 10 μM SB-431542 (R&D Systems), 100 U/mL penicillin, and 0.1 mg/mL streptomycin. Enteroid growth medium was replaced every 2 to 3 days. Enteroids were passaged every 5 to 7 days by incubating in 0.25% trypsin in PBS with 0.5 mM EDTA (Gibco) solution for 3 minutes at 37°C, followed by mechanical dissociation. Enteroids between passage number 7 and 20 were used to seed the chips.

### Neonatal-Intestine-on-a-Chip culture.

Two chambered microfluidic chips were obtained from Emulate Inc. and treated as previously described in detail (16), with minor modifications. Briefly, chips were activated following the manufacturer’s instructions using ER1 and ER2 solutions (Emulate Inc.). The top chamber of the chips was coated with 200 μg/mL type IV collagen (Sigma-Aldrich) and 100 μg/mL Matrigel growth factor–reduced matrix in PBS (Gibco), while the bottom chamber was coated with 200 μg/mL type IV collagen and 30 μg/mL fibronectin (Sigma-Aldrich) in PBS to facilitate cellular adhesion and mimic the composition of the extracellular matrix. The chips were then placed in a humidified incubator at 37°C and 5% CO_2_ overnight. Cultured neonatal human enteroids derived from human neonatal ileal tissue as described above in *In vitro culture of human neonatal enteroids* were dissociated, and resulting enteroid fragments were resuspended at a density of 6 × 10^6^ cells/mL in chip expansion media: 50% vol/vol mixture of Advanced DMEM/F12 and L-WRN with 10 μM Y-27632, 10 μM SB-431542, 10 nM human [Leu^15^]-gastrin I (Sigma-Aldrich), 500 nM A83-01 (Sigma-Aldrich), 10 mM HEPES, 100 U/mL penicillin, 0.1 mg/mL streptomycin, 1× B-27 Supplement (Gibco), 1× N-2 Supplement (Gibco), 1 mM *N*-acetylcysteine (Sigma-Aldrich), 50 ng/mL murine epidermal growth factor (EGF; PeproTech), and 10 mM nicotinamide (Sigma-Aldrich). The top channel of the chip was loaded with 30 μL of resuspended cells (~180,000 cells per chip) and maintained at 37°C overnight. The following day, seeded chips were washed with prewarmed expansion media to remove unattached cells. To provide a continuous flow of media across the epithelial cells attached to the chip, continuous perfusion with expansion media was initiated through the top and bottom channels with a flow rate of 30 μL/h. This recapitulates the flow across the epithelium that occurs in vivo, with movement of intraluminal contents across the intestinal epithelium on the apical side and perfusion of the cells provided by blood flow through underlying vasculature on the basolateral side. Once the neonatal epithelium reached confluence, repeated peristaltic-like membrane contractions were initiated at 10% strain and 0.2 Hz. This mimics the intestinal peristalsis that occurs in vivo.

Upon appearance of villus-like axes (day 6), human small intestinal microvascular endothelial cells (HIMECs; Cell Biologics) were resuspended at a density of 6 × 10^6^ cells/mL in Microvascular Endothelial Cell Growth Medium-2 (EGM-2MV, Lonza). The bottom channel of the chip was washed with EGM-2MV and loaded with 6 μL of HIMEC cell suspension (~36,000 cells per chip). The chips were inverted for 2 hours at 37°C to facilitate attachment of the HIMECs to the semipermeable membrane. After adhesion of HIMECs to the membrane, continuous flow at 30 μL/h was reinitiated with expansion media and EGM-2MV to the epithelial and endothelial channels, respectively.

### Bacterial culture.

An aliquot of intestinal bacteria isolated from a patient with severe, surgical NEC was obtained as previously described (24). Prior to addition to the chips, these bacteria were grown from 10 μL of a frozen glycerol stock incubated in 2 mL Luria-Bertani (LB) broth (Sigma-Aldrich) at 37°C in a shaking incubator. The bacteria were then diluted in chip expansion media to 7 × 10^8^ CFU/mL in preparation for addition to the chip.

### NEC-on-a-Chip model.

Antibiotic-containing cell culture medium was removed from the media reservoirs 24 hours prior to bacterial inoculation and media reservoirs were subsequently rinsed with sterile PBS. Antibiotic-free expansion media and antibiotic-free EGM-2MV were added to the top and bottom channel reservoirs, respectively. Where indicated, 30 μL of intestinal bacteria (7 × 10^8^ CFU/mL) was added after 24 hours to the top channel of the chip (2.1 × 10^7^ CFU per chip). For experiments detailed in Figure 3, 1 × 10^6^ CFU per chip were added. The chips remained under static condition for 30 minutes to facilitate bacterial adherence to the apical side of the neonatal epithelium. The top channel was flushed with antibiotic-free expansion media to remove unattached bacteria and the chips were continuously perfused with antibiotic-free media at 30 μL/h. Chips were flushed at an increased rate of 1000 μL/h for 6 minutes every 24 hours to remove unattached bacteria.

### Intestinal permeability assessment.

To measure apparent paracellular permeability (P_app_), 50 g/mL Cascade Blue (3 kDa, Thermo Fisher Scientific) was added to the antibiotic-free expansion media in the top epithelial channel with a flow rate of 30 μL/hr. Every 24 hours, the effluents from the epithelial and endothelial channels were collected. The concentration of Cascade Blue that diffused across the membrane into the endothelial channel effluent was quantified with a Varioskan LUX multimode microplate reader (Thermo Fisher Scientific) with excitation at 400 nm and emission at 425 nm. The following formula was used to calculate P_app_ (cm/s) as in Kasendra et al. (16): P_app_ = (*Endothelial_Output_* × *Flow Rate*)/(*Epithelial_Input_* × *Surface Area*), where *Endothelial_Output_* is the concentration (mg/mL) of Cascade Blue from the endothelial channel effluent, *Flow Rate* is the fluid flow rate (mL/s) of the media through both channels, *Epithelial_Input_* is the concentration (mg/mL) of Cascade Blue originally added to the epithelial channel media, and *Surface Area* is the area (cm^2^) of the channel where coculture occurs.

### RNA-seq and analysis.

Bulk RNA-seq was performed on small intestine epithelial cells obtained from the NEC-on-a-Chip and control chips by the Genome Technology Access Center (GTAC) at the Washington University School of Medicine in St. Louis, as in Nolan et al. (34). Briefly, RNA was extracted using TRIzol (Thermo Fisher Scientific). Libraries were then generated by the GTAC using the Kapa RiboErase kit (Roche) per the manufacturer’s instructions. Samples were subsequently indexed and pooled. Sequencing was then performed on an Illumina NovSeq 6000 with 2 × 150 base pair paired-end reads. Illumina RTA 1.9 was used to perform basecalls and Illumina’s bcl2fastq was used for demultiplexing.

mRNA expression was analyzed using Partek Flow software. After removal of adapters, reads were aligned with the hg38 reference sequence with STAR 2.7.3a and quantified to Ensembl transcripts release 91 with an average coverage of 10×. Reads were normalized to counts per million. Features containing fewer than 10 reads were not included in the analysis. Total number of aligned reads, the total number of uniquely aligned reads, and features detected were analyzed to determine sequencing performance. Differential gene expression was compared between groups. The results were filtered with the inclusion of genes with *P* values less than or equal to 0.05 and a fold change greater than 2.0 in pairwise comparisons. The RNA-seq data have been uploaded to the NCBI Gene Expression Omnibus (GEO GSE226086).

### RNA isolation, reverse transcription, and qRT-PCR.

Epithelial RNA was isolated from the top channel of the chips by administration of TRIzol. cDNA was synthesized using QuantiTect Reverse Transcription Kit (Qiagen) per the manufacturer’s instructions. Quantitative real-time PCR (qRT-PCR) was performed using SsoAdvanced Universal SYBR Green Supermix (Bio-Rad) in conjunction with a CFX Connect Real-Time PCR Detection System (Bio-Rad). Primers used are shown in Table 1 and relative expression levels were normalized to *RPLO* as a housekeeping gene.

### Immunofluorescence.

Immunofluorescent staining was performed on fixed, paraffin-embedded neonatal ileal tissue. Briefly, neonatal tissue slides were deparaffinized in xylene, rehydrated in isopropanol, and boiled in antigen unmasking solution (Vector Laboratories). Slides were then blocked with 1% bovine serum albumin (BSA; VWR) and 10% normal donkey serum (NDS; Sigma-Aldrich) in 1× Tris Buffered Saline (Boston BioProducts) with 0.1% Tween 20 (Sigma-Aldrich) (TBST). Primary antibodies (Table 2) were diluted 1:100 in blocking solution and secondary antibodies (Life Technologies) were diluted 1:200 in blocking solution. Nuclear staining was performed using Hoechst 33342 dye (Invitrogen) and slides were mounted with Prolong Gold (Thermo Fisher Scientific). Slides were imaged on either a Zeiss Axio Observer.Z1 using an EC Plan-Neofluar 10× objective (Zeiss) with either a 1× tube-lens or 1.6× tube-lens optical variable adapter (Zeiss) or a white light laser Leica SP8X tandem scanning confocal microscope (Leica Microsystems) with a 40× 1.3 NA oil objective (Leica Microsystems). Images were processed using either ZEN pro, blue edition (v2.3, Zeiss) or LASX (Leica Microsystems) software.

Immunofluorescent staining of the chips was performed as previously described (16). In brief, chips were fixed in 4% paraformaldehyde (Alfa Aesar), permeabilized with 0.1% Triton X-100 (Sigma-Aldrich), and blocked in 10% NDS in PBS. Chips were stained with primary antibodies (Table 2) diluted in 5% NDS in PBS, and secondary antibodies diluted 1:200 in 5% NDS in PBS, and either Hoechst or DAPI. Where indicated, coronal cross sections of the chips were hand-cut with a razor blade at approximately 300 μm thickness. Coronal cross sections were then imaged in an imaging dish with PBS.

A white light laser Leica SP8X tandem scanning confocal microscope was used to image the chips with a 25× water objective (Leica Microsystems). Images were acquired every 0.5 μm in the *z*-plane to render 3D images. Images were generated and analyzed using LASX and Volocity (v6.3.5; Quroum Technologies) software. Where indicated, 3D deconvolution was performed using Huygens Essential, v19.10 (Scientific Volume Imaging).

### Light microscopy.

The epithelial structure of the top channel of the chip was assessed over time with brightfield microscopy using a Zeiss Axio Observer.Z1 in combination with either an EC Plan-Neofluar 10× objective or a Plan-Apochromat 10× objective (Zeiss) and either a 1× tube-lens or a 1.6× tube-lens optical variable adaptor (Zeiss). Live-cell, representative *z*-plane images were obtained at 24-hour time intervals at 37°C and 5% CO_2_. Images were processed using ZEN 2.3 pro, blue edition software.

### Statistics.

Statistical analyses with Mann-Whitney *U* test and 2-tailed, unpaired *t* tests were performed where indicated using GraphPad Prism software version 8.0. Outliers were identified using the ROUT method. Statistical significance was accepted at a *P* value of less than 0.05.

### Study approval.

Deidentified premature infant small intestine was obtained from infants undergoing surgical resection for NEC or for noninflammatory conditions (controls). All specimens were processed as discarded tissue with a waiver of consent approval of the Washington University in St. Louis School of Medicine Institutional Review Board (IRB protocol numbers 201706182 and 201804040).

## Author contributions

WEL, CJL, SEG, and M Good conceptualized the study. WEL, CJL, LSN, QG, LCF, JMR, SEG, RL, SSB, CAS, ANL, BM, PA, M Goree, MM, EH, DGP, and M Good developed the methodology and carried out the investigation. WEL and M Good wrote the original draft of the manuscript, which was reviewed and edited by all coauthors. CJL, LSN, LCF, and M Good acquired funding. M Good provided resources and supervised the study.

## Figures and Tables

**Table 2:**
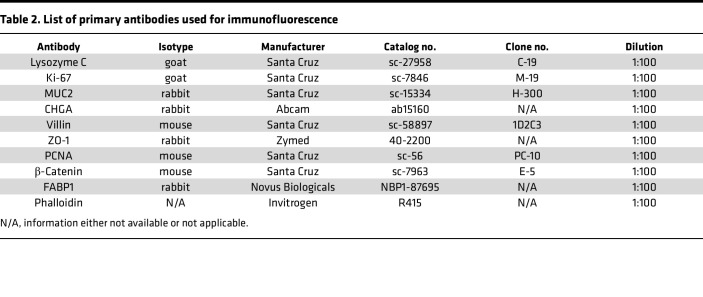
List of primary antibodies used for immunofluorescence

**Table 1:**
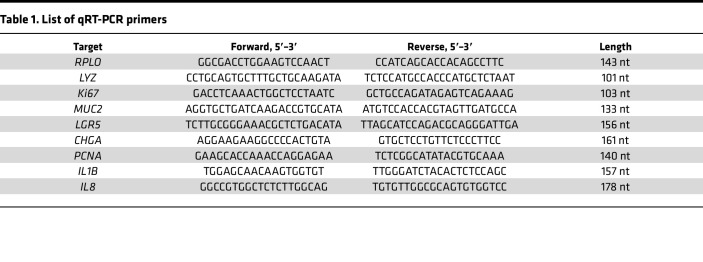
List of qRT-PCR primers

## Version 1 03/07/2023

In-Press Preview

## Version 2 04/24/2023

Electronic publication
